# Elimination Rates of Dioxin Congeners in Former Chlorophenol Workers from Midland, Michigan

**DOI:** 10.1289/ehp.1205544

**Published:** 2012-10-11

**Authors:** Lesa L. Aylward, James J. Collins, Kenneth M. Bodner, Michael Wilken, Catherine M. Bodnar

**Affiliations:** 1 Summit Toxicology, LLP, Falls Church, Virginia, USA; 2 The Dow Chemical Company, Midland, Michigan, USA

**Keywords:** dioxin, occupational exposure, toxicokinetics

## Abstract

Background: Exposure reconstructions and risk assessments for 2,3,7,8-tetrachlorodibenzo-*p*-dioxin (TCDD) and other dioxins rely on estimates of elimination rates. Limited data are available on elimination rates for congeners other than TCDD.

Objectives: We estimated apparent elimination rates using a simple first-order one-compartment model for selected dioxin congeners based on repeated blood sampling in a previously studied population.

Methods: Blood samples collected from 56 former chlorophenol workers in 2004–2005 and again in 2010 were analyzed for dioxin congeners. We calculated the apparent elimination half-life in each individual for each dioxin congener and examined factors potentially influencing elimination rates and the impact of estimated ongoing background exposures on rate estimates.

Results: Mean concentrations of all dioxin congeners in the sampled participants declined between sampling times. Median apparent half-lives of elimination based on changes in estimated mass in the body were generally consistent with previous estimates and ranged from 6.8 years (1,2,3,7,8,9-hexachlorodibenzo-*p*-dioxin) to 11.6 years (pentachlorodibenzo-*p*-dioxin), with a composite half-life of 9.3 years for TCDD toxic equivalents. None of the factors examined, including age, smoking status, body mass index or change in body mass index, initial measured concentration, or chloracne diagnosis, was consistently associated with the estimated elimination rates in this population. Inclusion of plausible estimates of ongoing background exposures decreased apparent half-lives by approximately 10%. Available concentration-dependent toxicokinetic models for TCDD underpredicted observed elimination rates for concentrations < 100 ppt.

Conclusions: The estimated elimination rates from this relatively large serial sampling study can inform occupational and environmental exposure and serum evaluations for dioxin compounds.

Epidemiologic studies often use current serum dioxin concentrations and pharmacokinetic models to estimate past workplace dioxin exposures. Risk assessments for dioxins also rely on estimates of elimination rates and pharmacokinetic models to translate between intakes and serum concentrations of these compounds. The validity of these models and approaches depends on the rate that dioxins are cleared from the body, as well as other physiologic factors.

We previously studied serum concentrations of 2,3,7,8 tetrachlorodibenzo-*p*-dioxin (TCDD) and other higher chlorinated dioxins in former 2,4,5-trichlorophenol (TCP) and pentachlorophenol (PCP) workers from Midland, Michigan (Collins et al. 2006). TCDD concentrations above background levels were measured in the serum of some TCP workers and concentrations of the higher chlorinated dioxins were elevated in PCP workers. These concentrations were used to estimate past exposures to TCDD and higher chlorinated dioxins including 1,2,3,4,7,8-hexachlorodibenzo-*p*-dioxin (14HxCDD), 1,2,3,6,7,8-hexachlorodibenzo-*p*-dioxin (16HxCDD), 1,2,3,7,8,9-hexachlorodibenzo-*p*-dioxin (19HxCDD), 1,2,3,4,7,8,9-heptachlorodibenzo-*p*-dioxin (HpCDD), and octachlorodibenzo-*p*-dioxin (OCDD) (Collins et al. 2006). Mortality rates among chlorophenol workers by past exposure levels derived in this way were reported previously (Collins et al. 2009a, 2009b).

In the present study we resampled a subset of chlorophenol workers with previous serum concentration measurements to estimate elimination rates of these dioxins. Previous studies have provided a wide range of average half-life estimates for TCDD, from 6.1 years to 11.3 years in adults (Flesch-Janys et al. 1996; Milbrath et al. 2009; Wolfe et al. 1994). There are fewer studies on the half-lives of the higher chlorinated dioxins found in former PCP workers (Flesch-Janys et al. 1996; Rohde et al. 1999). In addition, factors such as chloracne, age, amount of body fat, and smoking have been related to dioxin levels or elimination rates (Flesch-Janys et al. 1996; Milbrath et al. 2009; Wolfe et al. 1994). Finally, ongoing exposure to TCDD and other dioxin-like compounds occurs in the general population at low and declining levels through the presence of these compounds in the food chain (Lorber et al. 2009). Thus, a chlorophenol worker’s current dioxin levels reflect a combination of duration and concentration of past exposures; elimination rates, which may vary with age or body composition changes; current intake levels; and possibly other factors.

Here we present the results of a second serum sampling conducted on a subset of former chlorophenol workers included in a previous serum sampling study (Collins et al. 2006). We used several approaches to estimate first-order elimination rates for the dioxin congeners based on these serial sampling data, and we examined factors that might influence the observed elimination rates.

## Methods

*Participant selection.* The selection strategy called for drawing a stratified random sample of workers included in the previous 2004–2005 blood draw. Only people < 80 years of age on 15 October 2010 were eligible, because advanced age was found to limit mobility of the candidates and their ability to safely provide a sufficient blood sample. We used the 95th percentile of age-specific U.S. population concentrations for each congener identified in the National Health and Nutrition Examination Survey (NHANES; Centers for Disease Control and Prevention 2009) to define the upper ends of the background ranges. Nine strata were formed based jointly on previous serum levels of TCDD (high, > 50 ppt; moderate, 10–50 ppt; and background, < 10 ppt), representing workers in predominantly TCP processes, and OCDD (high, > 5,000 ppt; moderate, 1,000–5,000 ppt; and background, < 1,000 ppt), representing those with experience in PCP processes. We attempted to enroll participants representing each of these strata. Invitations to participate were sent to our target sample of 72 workers who were selected based on these congener concentration criteria.

*Questionnaire and data collection methods.* We conducted the serum analyses in 2010 using the same protocol, procedures, and laboratory as in the 2004–2005 blood draw (Collins et al. 2006). The serum samples were tested for 7 polychlorinated dibenzo-*p*-dioxins (PCDDs), 10 polychlorinated dibenzofurans (PCDFs), and 15 polychlorinated biphenyls (PCBs). Because previous studies of these workers indicated that workplace exposures increased concentrations of PCDDs—but not PCDFs or PCBs—we focused on the 7 PCDDs. However, we also calculated total TCDD toxic equivalency (TEQ) as a potency-weighted sum of 7 PCDD, 10 PCDF, and and 4 coplanar PCB congeners (PCBs 77, 81, 126, and 169) (Van den Berg et al. 2006). The study was conducted pursuant to approval and oversight by the Dow Human Subjects Review Board in Midland, Michigan. All study participants gave informed consent.

On the day of examination in the fall of 2010, participants donated blood and completed the same questionnaire originally administered during the 2004–2005 sampling effort (Collins et al. 2006). The questionnaire addressed dietary and smoking habits, work history, and recent changes in weight. Height, weight, and blood pressure were also measured. Diagnosis of chloracne during occupational exposure was extracted from workplace medical records; the criteria for chloracne diagnosis were defined previously by Bond et al. (1990).

*Elimination rates.* Changes in measured concentrations of persistent compounds such as PCDDs and PCDFs reflect not only intrinsic elimination rates but also any ongoing intakes of the compounds and changes in the volume of distribution, in this case, the volume of body lipids (Bartell 2012; Ritter et al. 2009, 2011). If current body concentrations are high relative to steady-state concentrations associated with current background intake rates, apparent elimination rates will approximate intrinsic elimination rates (Bartell 2012; van der Molen et al. 1998). If, on the other hand, background intakes are high compared with current body concentrations, the estimated apparent elimination rate will be lower than the intrinsic rates. Robust data regarding ongoing congener intake rates were not available, so the elimination rates were first calculated based on net rate of change of the congeners, which reflects both intrinsic elimination rates and unmeasured intake rates, termed “apparent” elimination rates.

We calculated apparent elimination rates in two ways: based on changes in concentration, or based on changes in estimated body amounts. For the former approach, concentrations of congener *i* in 2005 and 2010 (*C_i_*_,2005_ and *C_i_*_,2010_, respectively) and time between sampling dates (Δ*t*), were used to estimate the elimination rate (*k_i_*):


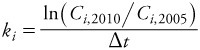
[1]

Concentrations of lipophilic compounds may change in response to changes in the volume of distribution (increase or decrease in amount of body fat), even in the absence of any elimination or additional exposure (Charlier et al. 2002; Chevrier et al. 2000; Hue et al. 2006; Imbeault et al. 2001; Pelletier et al. 2002; Walford et al. 1999). Thus, we also estimated the rate of change in estimated whole body mass of each congener. For the approach based on changes in estimated mass of dioxins in the body, the congener concentrations in 2005 and 2010 were replaced by the estimated amounts, *A*, of congener *i* in the body:


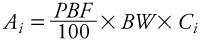
[2]

where *PBF* is percent body fat estimated using an age-, sex-, and body mass index (BMI)-specific formula, which has been validated in adults over a range of ages including those of the participants in this study (Deurenberg et al. 1991), *BW* is body weight in kilograms, and *C_i_* is the serum lipid–adjusted concentration of congener *i*. This approach assumes that the compound is distributed solely in body lipid. The resulting amount, *A_i_*, calculated for each sampling time and congener was used in Equation 1 in place of concentration for calculating the apparent whole-body mass-based elimination rate. Apparent elimination rates calculated using these two approaches were derived for each individual for each dioxin congener and for summed dioxin toxic equivalents [TEQ; WHO (World Health Organization) toxic equivalency factors (van den Berg et al. 2006)].

Elimination half-lives (*t_1/2_*) corresponding to the estimated elimination rates, (*k*) were calculated for some comparisons with previous literature:


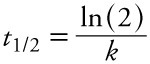
[3]

We investigated potential associations with covariates that could influence the apparent elimination rate. Regression variables included age; serum lipid–adjusted congener concentration in 2005; chloracne diagnosis (ever vs. never); BMI in 2010; smoking in 2010 (current vs. former or nonsmokers); former employment on either the TCP or PCP manufacturing processes or both; and change in estimated body fat volume between sampling points. We visually inspected the estimated apparent elimination rates for outliers and omitted these from regression analyses. We identified covariates associated with the estimated elimination rates with *p*-values of ≤ 0.1. All statistical analyses were conducted using STATA IC 10 software (StataCorp., College Station, TX).

In previous studies and modeling of elimination of persistent lipophilic chemicals, apparent elimination rates—calculated using Equation 1, which neglects ongoing intakes—declined as concentrations approached background or steady-state concentrations (Bartell 2012; van der Molen et al. 1998). Increased elimination rates have been found to be associated with highly elevated serum TCDD concentrations, potentially resulting from the induction of hepatic enzymes that facilitate faster elimination (Aylward et al. 2005; Emond et al. 2006). For these two reasons, we hypothesized that apparent elimination rates might be positively associated with congener concentrations. Therefore, in addition to including the measured concentration in 2005 as a continuous variable in the regressions described above, we conducted a categorical evaluation by comparing the estimated mass-based elimination rates for the key occupationally related congeners in those participants with serum concentrations in 2004–2005 in excess of the NHANES age-specific 95th percentile (Patterson et al. 2009) to the rates in individuals without elevated concentrations. Using a one-sided Fisher’s exact test, we conducted a nonparametric test of equality of medians to test the null hypothesis that medians in the two groups were not different.

*Inclusion of plausible background exposures.* As noted above, the apparent first-order elimination rates calculated using Equation 1 do not take into account any ongoing background exposure. Changes in concentration or estimated mass of compound in the body reflect the net effect of both elimination and any ongoing exposures between sampling time points. Under a first-order model, the amount of compound in the body in 2010 (*A_2010_*) is a function of both the intrinsic elimination rate, *k_int_*, and the ongoing exposure rate between sampling time points:


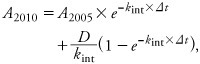
[4]

where *D* is the yearly dose rate in nanograms per year, and *A_2005_* is the starting amount in 2005. As described by Ritter et al. (2011), intrinsic elimination rates can be estimated if ongoing exposure levels are known or can be estimated.

Some data are available regarding estimated exposures to dioxin-like compounds in the United States. Lorber et al. (2010) estimated total TEQ intakes of 33.5 pg/day using databases of food-specific concentrations and estimates from other exposure pathways during the time period relevant to the sampling interval for the present study. However, no congener-specific background exposure estimates for the U.S. general population are available. As an approximation for the apportionment of TEQ intake among specific congeners, we used the distribution of these congeners in human serum.

Scott et al. (2008) presented the percent contribution of each dioxin-like compound to total TEQ in serum in biomonitoring data from NHANES data from 2001–2002. Although the TEQ proportion in serum would be expected to differ from the TEQ proportion in current dietary intake because of differential congener pharmacokinetics and historical exposure profiles, the proportions in human serum lipid are likely not too different from the proportions in animal fats, which are the primary contributors to human dietary exposure (Lorber et al. 2009). Thus, we used the estimated percent contribution of each dioxin congener to total TEQ in serum from Scott et al. (2008) to apportion estimated daily TEQ exposure from Lorber et al. (2010) among the dioxin congeners, and solved Equation 4 for intrinsic elimination rate, *k_int_*, for each congener and individual, assuming that each individual experienced the same congener-specific average daily exposures over the time period between measurements.

*Concentration-dependent models for TCDD.* Concentration-dependent elimination behavior for TCDD has been observed at high exposure levels, most obviously at serum lipid concentrations > 1,000 ppt (Aylward et al. 2005). Based on these observations and data on concentration-dependent distribution and elimination in laboratory animals, two toxicokinetic models reflecting this behavior have been published [our model (Aylward et al. 2005), and the Emond model as described by the U.S. Environmental Protection Agency (2012)]. Apparent elimination behavior in the range of TCDD serum lipid concentrations observed in the cohort examined here (up to ~ 150 ppt at the 2005 sampling time point) was simulated with both models by first applying a constant exposure level up to 63 years of age, then simulating the change in serum lipid concentration with no additional exposure to age 68 years (the mean ages of the study participants in 2005 and 2010, respectively). The exercise was conducted for a range of exposure levels (0.06–10 pg/kg-day), with resulting TCDD serum lipid concentrations ranging from approximately 3 to 150 ppt at modeled age 63 years. We entered the modeled serum lipid concentrations at ages 63 and 68 into Equation 1 to calculate the apparent first-order half-lives of elimination predicted by these models for comparison with the apparent elimination rates estimated from the serial sampling data for participants in this study.

## Results

Of the 72 eligible workers or former workers selected for the new blood draw in 2010, 56 (78%) agreed to participate. The total included 7 participants from the 2005 sampling who had serum TCDD levels in the “high” category, and 6 participants from the “high” serum OCDD category. There were no eligible candidates from the previous study (and therefore no participants) with both high TCDD and high OCDD values, although all other TCDD/OCDD level combinations were represented. Characteristics of the study population are presented in Table 1. Participants in 2010 were on average older than 68 years, with a relatively low proportion of smokers. The overall average BMI was relatively stable between the two sampling periods for these 56 individuals, but we observed large differences among the participants in change in BMI and change in estimated body fat volume.

**Table 1:** Demographic information and chlorophenol work assignments for 56 Midland study participants (all male) with repeated blood sampling for dioxins.

Parameter	2004–2005	2010
Age at sampling [mean ± SD (range)]	63.3 ± 7.1 (47.9–75.0)	68.5 ± 7.0 (54.3–80.0)
Smoking [n (%)]	8 (14.3)	7 (12.5)
BMI (mean ± SD)	32.0 ± 4.3	32.1 ± 4.6
Percent change in BMI [mean ± SD (range)]	0.7 ± 7.2 (–21.0 to 19.7)		
Percent change in estimated body fat volume [mean ± SD (range)]	3.9 ± 14.6 (–38.4 to 42.0)		
Total ever diagnosed with chloracne [n (%)]	15 (26.8)		
Total worked in TCP process only [n (%)]	30 (53.6)		
Total worked in PCP process only [n (%)]	17 (30.4)		
Total worked in both TCP and PCP processes [n (%)]	9 (16.1)		

Mean concentrations of each of the dioxin congeners declined between 2004–2005 and 2010 (Table 2). The percentage of participants with measured concentrations of each congener in 2004–2005 that exceeded the 95th percentile from the 2003–2004 NHANES data for ages > 60 years ranged from 34% (19HxCDD) to 68% (TCDD).

**Table 2:** Concentrations of analyzed dioxin congeners (pg/g lipid) in 56 Midland study participants with repeated blood samples.

Congener	2004–2005		2010		Mean percent change	NHANES (2003–2004) [P50, P95]b	> NHANES (2003–2004) P95 [n (%)]c
	Percent ND^a^	Mean ± SD	Percent ND^a^	Mean ± SD			
TCDD	0.0	21.1 ± 30.0	0.0	12.9 ± 19.6	–39	< LOD, 7.9	38 (67.9)
PeCDD	0.0	19.7 ± 11.3	0.0	14.2 ± 8.6	–28	8.0, 17.7	26 (46.4)
14HxCDD	0.0	16.0 ± 11.9	1.8	10.5 ± 7.9	–34	< LOD, 15.1	21 (37.5)
16HxCDD	0.0	136.6 ± 99.9	0.0	94.3 ± 70.8	–31	47.3, 104	29 (51.8)
19HxCDD	0.0	19.4 ± 14.5	3.6	11.3 ± 9.8	–42	< LOD, 16.2	19 (33.9)
HpCDD	0.0	159.9 ± 161.8	0.0	87.1 ± 83.3	–46	45.6, 132	21 (37.5)
OCDD	0.0	1919.6 ± 2121.0	0.0	1124.7 ± 1232.0	–41	370, 1,180	29 (51.8)
TEQd		71.4 ± 36.8		48.6 ± 26.3	–32	< LOD, 63.2	27 (48.2)
Abbreviations: LOD, limit of detection; ND, not detected. The NHANES 50th and 95th percentiles (P50 and P95, respectively) from the 2003–2004 cycle for ages ≥ 60 years are provided for comparison, along with the number and percent of Midland study participants with 2004–2005 concentrations > NHANES P95. aPercent of participants with no detected concentration of the congener at the specified sampling time point. bNHANES 2003–2004 P50 and P95 for persons ≥ 60 years of age (Patterson et al. 2009); LODs were variable in this survey. cIndividuals sampled in 2004–2005 having concentrations greater than the NHANES (2003–2004) P95 for ages ≥ 60 years. dWHO TEQ including 7 PCDD, 10 PCDF, and 4 PCB compounds (PCBs 77, 81, 126, and 169) (van den Berg et al. 2006).														

*Estimation of plausible background intake rates.* Table 3 shows the average proportion of total serum TEQ represented by each dioxin congener in the U.S. general population from the NHANES 2001–2002 survey as reported by Scott et al. (2008). The corresponding apportionment of total estimated daily TEQ exposure of 33.5 pg TEQ/day (Lorber et al. 2010) is also shown in units of both TEQ and mass intake. We included the resulting estimates of daily intake of each dioxin congener in modeling using Equation 4 to estimate a congener-specific first-order intrinsic elimination rate for each individual, assuming constant exposure over the time period between sampling (Ritter et al. 2011).

**Table 3:** Plausible estimated intake of dioxin congeners during the period between sampling.

Congener	TEF	Percent of serum TEQ^b^	Estimated intakes^a^		
			pg TEQ/day	pg/day	ng/year
TCDD	1	12.4	4.1	4.1	1.5
PeCDD	1	18.1	6.1	6.1	2.2
14HxCDD	0.1	2.3	0.8	7.7	2.8
16HxCDD	0.1	20.1	6.7	67.3	24.6
19HxCDD	0.1	2.7	0.9	8.9	3.3
HpCDD	0.01	2.3	0.8	77.7	28.4
OCDD	0.0003	0.6	0.2	673.1	245.7
aBased on apportionment of estimated 33.5 pg TEQ/day average total TEQ intake from Lorber et al. (2010). We used the proportion of human serum lipid TEQ in NHANES 2001–2002 accounted for by each dioxin congener (Scott et al. 2008) to apportion total TEQ intake among the dioxin congeners. bPercent of serum lipid TEQ concentration attributable to the specified congener from the NHANES 2001–2002 data set (Scott et al. 2008).					

*Elimination rates for dioxin congeners.* Table 4 presents the medians and interquartile ranges of the estimated first-order elimination half-lives calculated from elimination rates on the basis of change in concentration, change in estimated body mass, and change in mass with inclusion of ongoing exposures as presented in Table 3. Mean or median half-life estimates from two previous studies (Flesch-Janys et al. 1996; Rohde et al. 1999) are also presented. The estimated elimination half-lives from the present study based on changes in mass are slightly longer than those based on concentration. This is consistent with the small overall increase in body fat volume estimated for this population. Average estimated body fat volume increased both because the age-related formula used to estimate body fat predicts a greater proportion of body fat with increasing age (Deurenberg et al. 1991) and because average BMI increased slightly in this population between the first and second sampling period. The trend of increasing volume of body fat would tend to increase the apparent elimination rate based on changes in concentration because the greater fat volume provides a greater volume of distribution for the lipophilic compounds, resulting in an apparent decrease in concentration even in the absence of any elimination.

**Table 4:** Estimated half-lives of elimination (years) for selected dioxin congeners from serial serum samples of 56 Midland study participants.

Congener	Elimination half-lives [median (P25–P75)]			Flesch-Janys et al. 1996 (median)a	Rohde et al. 1999 (mean)b
	Apparent, concentration based	Apparent, mass based	Estimated intrinsic		
TCDD	6.5 (5.0–8.2)	7.0 (5.3–8.9)	6.5 (5.0–8.2)	7.2	8.7
PeCDD	10.7 (7.1–20.1)	11.6 (8.6–16.9)	10.7 (8.2–15.9)	15.7	13.9
14HxCDD	8.1 (5.8–12.2)	8.2 (6.0–13.8)	7.0 (5.6–11.3)	8.4	13.9
16HxCDD	10.1 (6.5–16.6)	11.0 (7.7–15.4)	9.0 (7.0–13.2)	13.1	11.6
19HxCDD	6.2 (5.0–8.9)	6.8 (5.5–9.0)	6.3 (5.2–8.4)	4.9	7.7
HpCDD	7.0 (5.3–9.9)	7.6 (5.8–11.2)	6.7 (5.2–9.0)	3.7	4.3
OCDD	7.8 (6.1–10.8)	8.3 (6.6–12.0)	7.3 (5.8–10.5)	6.7	8.7
TEQc	9.0 (6.7–13.1)	9.3 (7.5–15.1)	8.7 (6.9–12.3)
P25–P75, 25th–75th percentiles. Estimates were calculated on the basis of change in concentration, change in estimated mass of the congener in the body, or change in body mass after accounting for plausible background intake (Table 3). Half-lives from two previous studies, calculated from change in concentration, are included for comparison. an = 26 to 48, depending on the congener; mean age was 48.7 years at first sampling (range, 32–79 years). bn = 6; ages 41–73 years. cWHO TEQ including 7 PCDD, 10 PCDF, and 4 PCB compounds (PCBs 77, 81, 126, and 169) (van den Berg et al. 2006).										

Estimated mass-based half-lives derived using Equation 4 with the inclusion of plausible background exposures, as described in Table 3 (referred to as estimated intrinsic rates in Table 4), are approximately 10–20% shorter for each dioxin congener than the mass-based apparent half-lives calculated without consideration of the background exposure. We investigated the impact of alternative assumptions regarding these background intake rates. Doubling the estimated intakes resulted in another 10–20% decrease in estimated half-lives, whereas halving the estimated intakes pushed the half-life estimates closer to the mass-based apparent half-lives calculated without considering background intakes (data not shown).

The results of the regression analysis of factors potentially influencing the apparent elimination rates for the occupationally related congeners are presented in Table 5. The concentration-based apparent elimination rates were positively associated with increasing fat mass for most of the congeners and TEQ. Because these compounds are lipophilic, increasing fat mass results in an increase in volume of distribution and a reduction in concentration, even in the absence of any elimination. When the regressions were conducted on the mass-based apparent elimination rates (without accounting for ongoing intakes), the positive associations with change in fat mass disappeared or became slightly negative. This supports the use of the mass-based elimination rates to account for the impact of change in volume of distribution in these analyses.

**Table 5:** Parameter coefficients, SEs, and statistical significance for the multiple regression of concentration- and mass-based apparent elimination rates of dioxin congeners on potential determinants in 56 Midland study participants with serial blood samples.

Congener	Regression coefficient (SE)					
	2005 concentration (ppt)	Chloracne (ever vs. never)	Age, (years)	2010 BMI	Smoker in 2010 (current vs. nonsmoker)	Increase in fat mass (%)
Concentration-based apparent rates						
TCDD	–0.0004 (0.0002)**	NS	NS	NS	NS	0.0008 (0.0004)**
PeCDD	0.001 (0.0004)**	NS	NS	–0.002 (0.001)*	0.038 (0.014)**	0.0012 (0.0003#
14HxCDD	NS	0.029 (0.016)*	NS	–0.0032 (0.0017)*	0.082 (0.023)#	0.0014 (0.0005)**
16HxCDD	NS	NS	NS	NS	NS	0.0015 (0.0004)#
19HxCDD	NS	NS	NS	NS	NS	NS
HpCDD	NS	NS	NS	NS	NS	0.001 (0.0005)*
OCDD	NS	NS	NS	NS	NS	NS
TEQ	NS	NS	NS	NS	NS	0.0013 (0.0003)#
Mass-based apparent rates						
TCDD	–0.0004 (0.0002)*	NS	NS	NS	NS	–0.0009 (0.0004)**
PeCDD	0.001 (0.0004)**	NS	NS	–0.002 (0.001)*	0.035 (0.014)**	NS
14HxCDD	NS	NS	NS	–0.0034 (0.0016)**	0.078 (0.022)#	NS
16HxCDD	NS	NS	NS	NS	NS	NS
19HxCDD	NS	NS	NS	NS	NS	NS
HpCDD	NS	NS	NS	NS	NS	NS
OCDD	NS	NS	NS	NS	NS	–0.0013 (0.0005)**
TEQ	NS	0.018 (0.01)*	NS	NS	NS	NS
NS, not significant (p > 0.1). One outlier each was identified visually for elimination rate estimates for TCDD, 14HxCDD, HpCDD, OCDD, and TEQ; two outliers were identified for PeCDD and 16HxCDD. All together, six individuals were omitted from one or more congener-specific regressions. *p < 0.1. **p < 0.05. #p < 0.01.						

None of the other parameters investigated was associated with the estimated elimination rates across all congeners. Age was not associated with elimination rate for any congener, contrary to a previous review of elimination rates for dioxin-like compounds (Milbrath et al. 2009). The difference in findings may be due to the relatively older age of our population (Milbrath et al. included data on young adults and children) and the relatively narrow age range included. Elimination rate was negatively associated with the measured TCDD concentration in 2005 and positively associated with the PeCDD concentration in 2005. However, for both congeners, the magnitude of the observed association was slight. The elimination rates for PeCDD and 14HxCDD were positively associated with smoking and negatively associated with BMI in 2010, but these factors were not significant (*p* < 0.1) for any other congeners. In all cases, the observed associations were of relatively low magnitude. We observed no associations between elimination rates and indicator variables for former employment on the TCP or PCP processes (data not shown).

**Figure 1:**
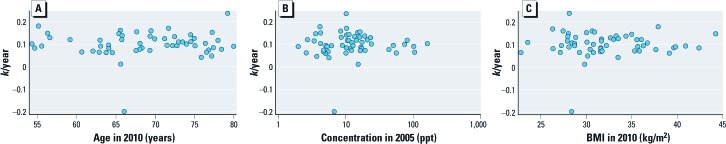
Scatter plots of the calculated mass-based apparent annual elimination rates (*k*) for TCDD among 56 subjects with serial blood samples versus (*A*) age in 2010, (*B*) concentration in 2005, and (*C*) BMI in 2010. One outlier with significant negative elimination (i.e., increase in concentration between the two sampling time points) is obvious in each scatter plot; this individual was omitted from the regression analyses for TCDD.

Figure 1 presents scatter plots of the mass-based apparent elimination rates for TCDD as a function of age in 2005, concentration in 2005, and BMI in 2010. These scatter plots illustrate that, with the exception of one outlier, the distribution of observed elimination rates was relatively consistent across all of these variables, as demonstrated by the results of the regression analyses. Examination of the scatter plots for the other congeners in this analysis show similar patterns (data not shown).

**Figure 2:**
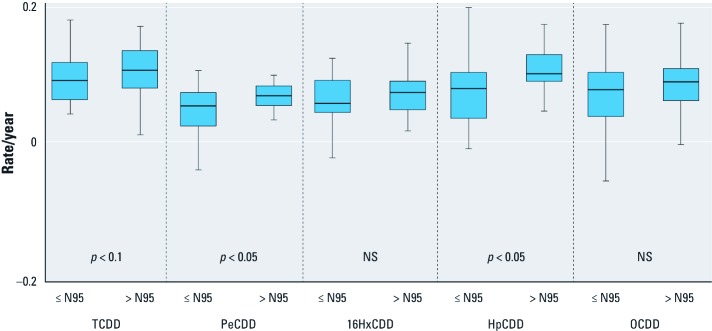
Distribution of mass-based apparent elimination rates for selected PCDD congeners. Rates are reported for each congener for participants with serum lipid concentrations in 2004–2005 that are less than or equal to (≤ N95) or greater than (> N95) the NHANES 95th percentile for persons ≥ 60 years of age. Boxes extend from the 25th to the 75th percentile, horizontal bars represent the median, and whiskers extend 1.5 times the length of the interquartile range above and below the 75th and 25th percentiles; outliers were not included. *p*‑Values represent one-sided Fisher’s exact test of equality of medians. NS, not significant.

We also examined potential influence of starting concentration on a categorical basis. Figure 2 presents box plots of the distribution of calculated mass-based elimination rates for the occupationally related congeners, stratified by whether the participant had a measured concentration in 2004–2005 in excess of the age-specific NHANES 95th percentile. The medians of the estimated mass-based apparent elimination rates in individuals in the elevated concentration groups were higher for three of the five major congeners (TCDD, PeCDD, and HpCDD). Similarly, the lower end of the range of elimination rates across individuals approached zero or was negative for four of the five congeners for participants in the lower concentration groups; that is, estimated body mass of the congener was unchanged or increased between the first and second sampling points for some individuals. These observations are consistent with the idea that as concentrations approach steady state with intake rates, the apparent elimination rates will decrease (Bartell 2012; van der Molen et al. 1998).

**Figure 3:**
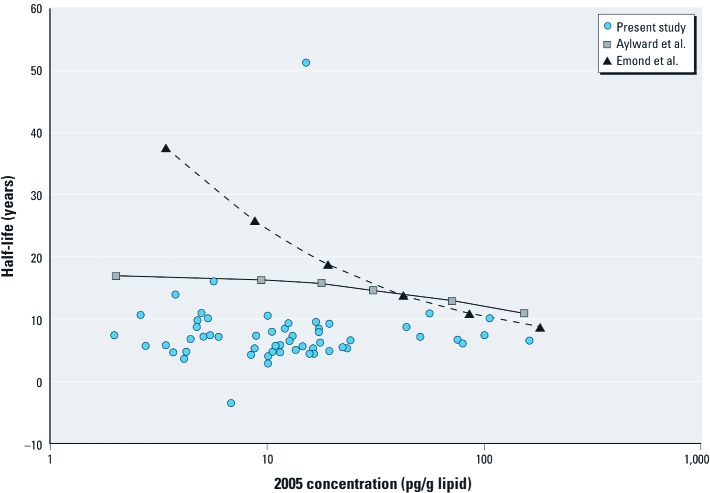
Mass-based apparent elimination half-lives for TCDD estimated in participants in the present study shown with the predicted apparent mass-based elimination half-lives from two concentration-dependent toxicokinetics models for TCDD: Aylward et al. (2005) and Emond et al. (2006). The two concentration-dependent models were run at constant intakes to the specified concentrations at 63 years of age, then intake was set to zero and concentrations recorded again at age 68 years. Mass-based apparent elimination rates were calculated using Equation 1 to allow for comparison with the participant data in the present study.

*Comparison to concentration-dependent models.* Calculated apparent first-order elimination half-lives for TCDD predicted by the two available concentration-dependent models are illustrated in Figure 3, along with the mass-based apparent elimination rates estimated in the present study. The predicted elimination half-lives from the concentration-dependent models are longer than the central tendency of the apparent elimination half-lives estimated for workers with serum lipid TCDD concentrations < 100 ppt (100 pg/g lipid) in 2005, but begin to approach the estimated apparent half-lives for workers with starting TCDD concentrations above that range. The concentration-dependent models presented here assumed no ongoing exposure between 63 and 68 years of age; inclusion of the estimated ongoing background exposure would increase the model-predicted half-lives, resulting in further disparity from the half-lives estimated based on measured concentration changes using mass-based apparent elimination rates for workers with starting concentrations < 100 ppt. These data and comparisons suggest that concentration-dependent model predictions for TCDD in the range of environmentally relevant exposures should be interpreted with caution; simple first-order models using the estimated half-lives based on data from this study and previous studies may prove more reliable in relating exposures to serum lipid concentrations < 100 ppt serum lipid.

## Discussion

This study provides an unusually large data set with two measurements of dioxin congener concentrations in blood from individuals over a period of approximately 5 years. This population of former workers includes individuals with elevated concentrations of higher chlorinated PCDD compounds resulting from historical occupational exposures. A large portion of the workers (27%) developed chloracne from these exposures. The data set also includes key information on body weight and height, as well as changes in body weight during the period between sampling. As a result, this study provides a unique and unusually large data set for examining the apparent rates of elimination and factors affecting those rates.

The estimated apparent elimination rates for chlorinated dioxin congeners in this population are generally similar to the elimination rates previously estimated by others (Table 4; Flesch-Janys et al. 1996; Rohde et al. 1999). The slightly longer half-life estimated for HpCDD in the present study may be due to the lower concentrations present in this population, if those concentrations are closer to steady state with ongoing intakes (Bartell 2012). The median HpCDD concentration reported by Flesch-Janys et al. (1996) at the first sampling time point was 641 pg/g lipid, whereas in the present study, the 2004–2005 median concentration was 88.6 pg/g lipid. If the concentrations in the present study are closer to those consistent with steady state with current background intake rates, the apparent half-life of elimination will be longer (Bartell 2012; van der Molen et al. 1998). In this respect, the estimates for elimination half-life reported by Flesch-Janys et al. (1996) may be more reliable as estimates of “intrinsic” elimination rates, but only if the ongoing intake rates for individuals in that study were relatively low compared with the measured concentrations in those individuals (Bartell 2012). Similar considerations influence interpretation of the more rapid elimination rates for PeCDD observed in the present study compared with the previous studies.

The lack of any consistent age-related trend in elimination half-lives in the present study is at odds with the findings from Flesch-Janys et al. (1996), in which elimination rates for each dioxin congener except OCDD were significantly and inversely related to the logarithm of age. We explored entry of age into the regression analyses in both linear and log scales but found no significant relationship in this population. As noted above, this may be due to the older and relatively narrow age range in this population. The age at the start of study ranged from 47.9 to 75 years, with a mean of 63.3 years, compared with to a wider range (32–79 years) and lower mean (48 years) for the study by Flesch-Janys et al. (1996); however, in that study, only one individual was > 65 years of age. Visual inspection of the data from Flesch-Janys et al. suggests that the relationship with age for TCDD in that study depends on the contrast between the youngest and oldest individuals in that group; little or no trend with age is apparent in individuals > 55 years of age in that data set. The absence of younger individuals in our study is because of cessation of chlorophenol operations in 1980 and the timing of the blood draws 25–30 years later.

Previous studies of the Dow Midland TCP- and PCP-manufacturing workers (Collins et al. 2009a, 2009b) used half-life estimates from Flesch-Janys et al. (1996) to estimate occupational exposure to and serum concentrations of TCDD, mixed HxCDDs, HpCDD, and OCDD. Serum profiles were used as exposure measures for dose–response analyses (Collins et al. 2009a, 2009b). The similarity of current and previous half-life estimates from the Midland cohort support the general validity of assumptions underlying our dose-reconstruction efforts.

The interindividual variation in the estimated elimination rates for each of the congeners generally encompassed an approximately 2-fold increase in apparent half-life from the 25th to the 75th percentile. This variation is likely a result of many contributing factors that were not assessed here, including individual differences in intrinsic metabolism and elimination efficiency and differences in dietary habits, which could lead to long-term interindividual variation in ongoing intake rates. Other sources of variation or uncertainty include imprecision in the methods we used to estimate body fat mass and inherent imprecision in analytical serum dioxin measurements, which is often in the range of 10–20%.

The data set presented here provides an unusually rich source of information about elimination rates of dioxin compounds resulting from occupational exposures and that are still present at elevated levels. However, because our data also extend into the range of concentrations observed in the general population, they have relevance to assessment of exposures at environmentally relevant concentrations. The continuing overall decline of all congeners in the serum of the Midland workers supports the conclusion that intakes continue to be lower than historical environmental exposure levels in Michigan, consistent with nationally observed trends (Patterson et al. 2009). The data on apparent elimination rates and potential impacts of smoking, body fat levels, and changes in body fat levels presented here can assist other evaluations, dose reconstruction, and risk assessment efforts both for populations with elevated exposures and for the general population.
